# Phased T2T genome assemblies facilitate the mining of disease-resistance genes in *Vitis davidii*

**DOI:** 10.1093/hr/uhae306

**Published:** 2024-11-06

**Authors:** Yuanyuan Luo, Zhenya Liu, Zhongxin Jin, Peng Li, Xibei Tan, Shuo Cao, Xu Wang, Zhongqi Liu, Xiaoya Shi, Siyang Huang, Liyuan Gu, Xiucai Fan, Jianfu Jiang, Lei Sun, Yongfeng Zhou, Chonghuai Liu, Xiaodong Xu, Zhiyao Ma, Ying Zhang

**Affiliations:** Zhengzhou Fruit Research Institute, Chinese Academy of Agricultural Sciences, Zhengzhou 450000, China; National Key Laboratory of Tropical Crop Breeding, Shenzhen Branch, Guangdong Laboratory of Lingnan Modern Agriculture, Key Laboratory of Synthetic Biology, Ministry of Agriculture and Rural Affairs, Agricultural Genomics Institute at Shenzhen, Chinese Academy of Agricultural Sciences, Shenzhen 518124, China; National Key Laboratory of Tropical Crop Breeding, Tropical Crops Genetic Resources Institute, Chinese Academy of Tropical Agricultural Sciences, Haikou 571101, China; Zhengzhou Fruit Research Institute, Chinese Academy of Agricultural Sciences, Zhengzhou 450000, China; Chuxiong Yunguo Agriculture Technology Research Institute, Chuxiong, China; Zhengzhou Fruit Research Institute, Chinese Academy of Agricultural Sciences, Zhengzhou 450000, China; National Key Laboratory of Tropical Crop Breeding, Shenzhen Branch, Guangdong Laboratory of Lingnan Modern Agriculture, Key Laboratory of Synthetic Biology, Ministry of Agriculture and Rural Affairs, Agricultural Genomics Institute at Shenzhen, Chinese Academy of Agricultural Sciences, Shenzhen 518124, China; National Key Laboratory of Tropical Crop Breeding, Shenzhen Branch, Guangdong Laboratory of Lingnan Modern Agriculture, Key Laboratory of Synthetic Biology, Ministry of Agriculture and Rural Affairs, Agricultural Genomics Institute at Shenzhen, Chinese Academy of Agricultural Sciences, Shenzhen 518124, China; National Key Laboratory of Tropical Crop Breeding, Shenzhen Branch, Guangdong Laboratory of Lingnan Modern Agriculture, Key Laboratory of Synthetic Biology, Ministry of Agriculture and Rural Affairs, Agricultural Genomics Institute at Shenzhen, Chinese Academy of Agricultural Sciences, Shenzhen 518124, China; National Key Laboratory of Tropical Crop Breeding, Shenzhen Branch, Guangdong Laboratory of Lingnan Modern Agriculture, Key Laboratory of Synthetic Biology, Ministry of Agriculture and Rural Affairs, Agricultural Genomics Institute at Shenzhen, Chinese Academy of Agricultural Sciences, Shenzhen 518124, China; National Key Laboratory of Tropical Crop Breeding, Shenzhen Branch, Guangdong Laboratory of Lingnan Modern Agriculture, Key Laboratory of Synthetic Biology, Ministry of Agriculture and Rural Affairs, Agricultural Genomics Institute at Shenzhen, Chinese Academy of Agricultural Sciences, Shenzhen 518124, China; Zhengzhou Fruit Research Institute, Chinese Academy of Agricultural Sciences, Zhengzhou 450000, China; Zhengzhou Fruit Research Institute, Chinese Academy of Agricultural Sciences, Zhengzhou 450000, China; Zhengzhou Fruit Research Institute, Chinese Academy of Agricultural Sciences, Zhengzhou 450000, China; Zhengzhou Fruit Research Institute, Chinese Academy of Agricultural Sciences, Zhengzhou 450000, China; National Key Laboratory of Tropical Crop Breeding, Shenzhen Branch, Guangdong Laboratory of Lingnan Modern Agriculture, Key Laboratory of Synthetic Biology, Ministry of Agriculture and Rural Affairs, Agricultural Genomics Institute at Shenzhen, Chinese Academy of Agricultural Sciences, Shenzhen 518124, China; National Key Laboratory of Tropical Crop Breeding, Tropical Crops Genetic Resources Institute, Chinese Academy of Tropical Agricultural Sciences, Haikou 571101, China; Zhengzhou Fruit Research Institute, Chinese Academy of Agricultural Sciences, Zhengzhou 450000, China; National Key Laboratory of Tropical Crop Breeding, Shenzhen Branch, Guangdong Laboratory of Lingnan Modern Agriculture, Key Laboratory of Synthetic Biology, Ministry of Agriculture and Rural Affairs, Agricultural Genomics Institute at Shenzhen, Chinese Academy of Agricultural Sciences, Shenzhen 518124, China; National Key Laboratory of Tropical Crop Breeding, Shenzhen Branch, Guangdong Laboratory of Lingnan Modern Agriculture, Key Laboratory of Synthetic Biology, Ministry of Agriculture and Rural Affairs, Agricultural Genomics Institute at Shenzhen, Chinese Academy of Agricultural Sciences, Shenzhen 518124, China; Zhengzhou Fruit Research Institute, Chinese Academy of Agricultural Sciences, Zhengzhou 450000, China; Chuxiong Yunguo Agriculture Technology Research Institute, Chuxiong, China; Zhongyuan Research Center, Chinese Academy of Agricultural Sciences, Henan, China

## Abstract

Grape is an important fruit crop, and its production faces significant threat from diseases, resulting in substantial economic loss. Wild grape relatives are valuable resources for the restoration of disease-resistance loci. However, available resistance loci in wild grape genomes remain largely unexplored. In this study, we assembled two phased genomes, including a high-resistant Chinese wild grape, *Vitis davidii* Föex, and a susceptible cultivar, *Vitis vinifera* L. cv. ‘Manicure Finger’. We detected a total of 36 688 structural variations (SVs), with the genes associated with heterozygous SVs showing an enrichment in allele-specific expression (ASE). Furthermore, we identified eight subgroups of R genes and found that 74.2% of R genes overlap with transposable elements (TEs). Among R genes, NBS-type genes exhibit higher expression profiles in the wild grape genome compared with those in the grape cultivar. Additionally, five specific NBS-type R gene clusters were identified in the wild grape genome that are absent in the cultivar. Through genetic mapping, we identified four quantitative trait loci (QTLs) associated with grape white rot resistance based on the *V. davidii* genome, within which six NBS-type R genes exhibit differential expression between wild and cultivated grapes. Overall, our study revealed the landscape of resistance genes in grape genomes, providing valuable genetic resources for further breeding programs.

## Introduction

Grape is one of the most important fruit crops in the world [1]. Recent research has provided genomic, historical, and geographical evidence of grape domestication and genetic diversity [2–4]. Disease is one of the significant threats to grape production, and severe infection often results in substantial economic loss [5,6]. The primary objective in breeding is to enhance stress tolerance, addressing challenges from the ongoing ‘arms race’ in plant–pathogen interaction [7–9]. The most popular varieties used to produce wine and table grapes belong to the European grape (*Vitis vinifera* L.) [10,11]. Although *V. vinifera* L. is adapted to a warm, dry climate during its growing season, their berries are highly susceptible to fungal diseases [12]. Exploring the diversity of global wild grape relatives is critical, especially for identifying more resistance loci. At the same time, wild grapes serve as valuable genetic resources for grape breeding [13], some of which have been incorporated into breeding programs to enhance both biotic and abiotic stress tolerance in modern cultivars [4,14–16].

Significant advances in sequencing technology have opened new avenues for deeper understanding of agronomic traits and resistance loci. In particular, high-fidelity (HiFi) long-reads sequencing have greatly improved the accuracy and reliability of genome assembly. A notable achievement in genome assembly is the telomere-to-telomere (T2T) approach, designed for assembling entire chromosomes. This approach provides a more comprehensive view of the complete genome, eliminating gaps and inaccuracies. For example, the genomes of several important grape varieties have been assembled (Table S1), including PN40024, Yan73 [17], Thompson Seedless [18], Cabernet Franc [19], Cabernet Sauvignon [20,21], Carménère [22], Chardonnay [23], Merlot [24], Nebbiolo [25], and Shine Muscat [26]. The assembly of the wild accessions has attracted wide attention in the grape community, such as that of *Vitis riparia* [27], *Vitis rotundifolia*, *Vitis acerfolia*, and *Vitis aestivalis* [28]. Additionally, genome assemblies for four Chinese wild grape species, including *Vitis amurensis* [29], *Vitis adenoclada* [30], *Vitis heyneana* [31], and *Vitis retordii* [16], have completed genome assembly.

Disease-associated resistance genes (R genes) often encode proteins recognizing specific pathogens, instigating defense responses, but the number of R genes vary across species [32]. For example, there are 50–100 nucleotide-binding domain and leucine-rich repeat (NLR) genes characterized in the genomes of *Carica papaya* [33], cucumber [34], and watermelon [35]. The number of NLR genes is highly variable across species and within the same family or genus [36–41]. Notably, bread wheat stands out with the identification of >2000 NLR-encoding genes [42]. In the rice [43] and lemon [44] genome studies, gapless genomic data has greatly expanded understanding of plant genome structure and facilitates the breeding of resistant varieties.

Grape cultivars generally exhibit reduced resistance to biotic and abiotic stresses compared to their wild relatives. Grape white rot is a major fungal disease affecting grape yield and quality. Most cultivated grapes, such as Manicure Finger, a cultivar of *V. vinifera,* have been shown to be susceptible to white rot [45]. *Vitis davidii* Föex exhibits considerable white rot resistance [46–48]. These observations underscore the potential and importance of tapping into *V. davidii* grape resources for sustainable viticulture. To fully understand the landscape of R genes and identify disease-resistance loci, we newly sequenced and constructed phase-resolved genomes of a wild grape *V. davidii* (0940) and a cultivar Manicure Finger. Our genomic analysis provides new insights into the evolution of R clusters and identifies new candidate resistance loci, thereby facilitating the development of resistant grape varieties.

**Figure 1:**
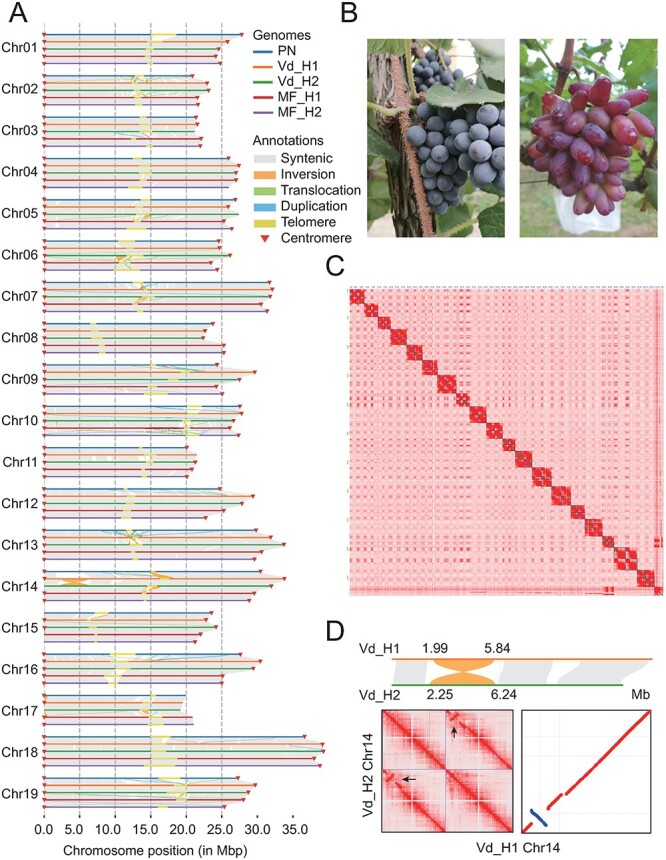
Grape genomes assembly and structural variations. A. Syntenic analysis results among five haplotypes (PNT2T, Vd_H1, Vd_H2, MF_H1, and MF_H2) and the telomeres and centromeres highlight the chromosomes. B. The grape images of a wild grape *V. davidii* (left) and a cultivated grape *V. vinifera* L*.* Manicure Finger (right). C. Hi-C interactions of chromosomes based on Vd assembly data. From top to bottom are chromosomes 1–19, respectively, and two squares are the same chromosome of Haplotype 1 (upper left) and Haplotype 2 (lower right), respectively. D. A 4.04-Mb heterozygous inversion on chromosome 14 from the Vd genome.

**Figure 2:**
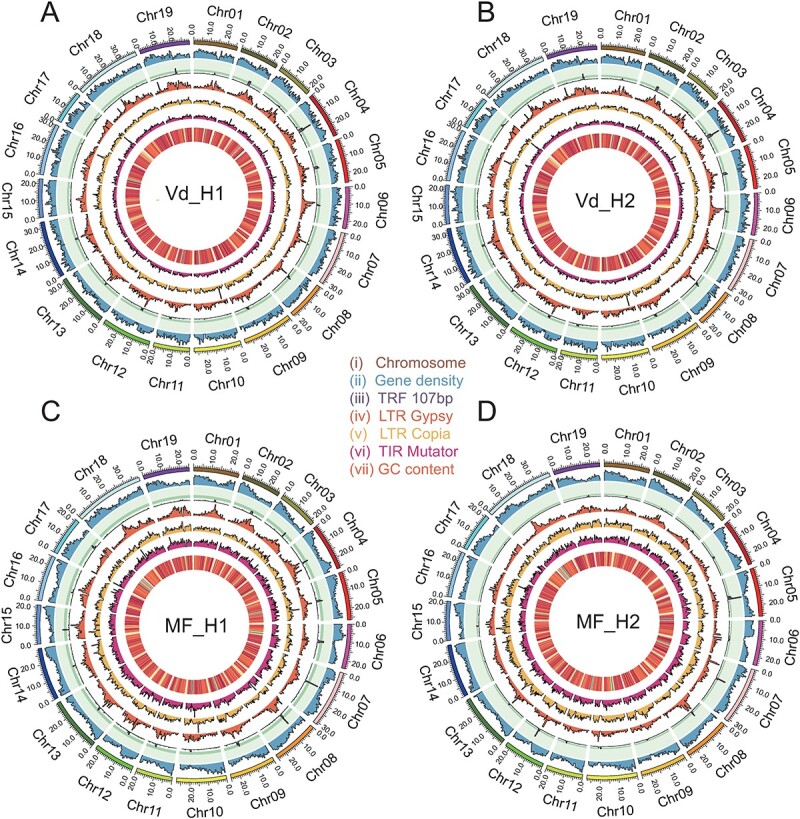
Grape genomes annotation. Genomic features of Vd_H1 (A), Vd_H2 (B), MF_H1 (C), and MF_H2 (D). Tracks from inside to outside are the length of the chromosomes, gene density, distribution of tandem repeats, distribution of LTR/Gypsy, distribution of LTR/Copia, distribution of TIR/Mutator, and GC content were calculated using a 50-kb window.

## Results

### Phase-resolved wild and cultivated grape genomes

We generated phase-resolved genome assemblies of a *V. davidii* Föex (namely Vd) grape and a *V. vinifera* L. Manicure Finger (namely MF) grape incorporating PacBio HiFi and high throughput chromosome conformation capture (Hi-C) sequencing data (Fig. 1b). Average of ~30 Gb of PacBio HiFi reads (55-fold coverage) and ~54 Gb of Hi-C paired reads (64-fold coverage) were generated for Vd and MF genomes, respectively (Table S2). We estimated heterozygosity for two grape genomes based on the k-mer method using HiFi reads. We found a lower heterozygosity in the wild grape genome (0.702%) than the cultivated grape (1.290%) genome (Fig. S1). Subsequently, we used HiFi and Hi-C reads for genome assembly based on a haplotype-resolved strategy (Fig. 1c, Fig. S2). As results, we obtained two haplotypes Vd_H1 and Vd_H2 (518.48 and 513.30 Mb) of the wild grape genome and two haplotypes MF_H1 and MF_H2 (489.74 and 486.96 Mb) of the cultivated grape genome (Fig. 1a). We found that contributions from seven chromosomes (2, 9, 12–14, 16, and 19) mainly expanded the size of the Vd genome relative to the MF genome, and the GC content of these haplotype assemblies ranges from 34.91% to 34.98% (Table S3). We identified the gene structures for each haplotype, with the number ranging from 31 368 to 33 153 (Fig. 2). The evaluation based on Benchmarking Universal Single-Copy Orthologs (BUSCOs) showed that the integrity of these haplotypes reached >98% (Table S4). The transposable element (TE) sequences account for a larger proportion in the Vd haplotypes (256.9 and 252.3 Mb) than in the MF haplotypes (235.8 and 232.3 Mb) (Table 1, Table S5). Our analysis found that TEs explain 74.46% of the increase in genome size. Therefore, TEs constitute a significant portion of grape genomes and contribute to the expansion of grape genome size. In addition, we identified centromeres of all haplotypes by utilizing a previously published 107-nucleotide monomer [49] (Fig. 2, Table S6). The comparative genomic analysis showed that the position of the centromere was conserved, but there were differences in the number of repeats. For example, the length of centromere in chromosome 1 of haplotype Vd_H1 (1.86 Mb) was two times larger than the haplotype MF_H1 (0.82 Mb). Through the analysis of ‘TTTAGGG/CCCATTT’ distribution, we identified the telomere positions on each haplotype: Vd_H1, 17/19; Vd_H2, 16/19; MF_H1, 17/19; and MF_H2, 17/19 (Fig. 1a). Overall, our analyses provided two high-quality phase-resolved grape genomes for further utilization in grape breeding.

The PNT2T genome was the first T2T genome of the grape [50]. Using PNT2T as a reference, we constructed a genome-wide variation map including 3.24 million single nucleotide polymorphisms (SNPs) and 36 688 reliable structural variations (SVs), of which 52.4% were deletions (DELs) and 47.3% were insertions (INSs) (Fig. S3, Table S7). Additionally, we identified a 4.04-Mb inversion (INV) on chromosome 14 of the Vd genome, which was confirmed to exist on the chromosome through Hi-C reads validation (Fig. 1d). We genotyped these SVs and found that the number of heterozygous SVs in the MF genome (5095) is higher than the Vd genome (2171) (Fig. S4).

### The comparative genomics and SVs in grapes

**Figure 3:**
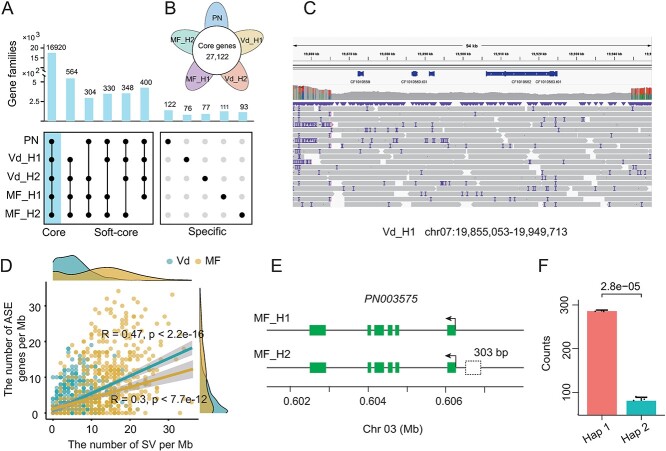
Variable genes and expression affected by SVs. A. The gene family in four newly assembled haplotypes and PNT2T genome. The core and variable (soft-core and specific) gene families were shown. B. The number of conserved genes in five haplotypes. C. An 82-kb heterozygous deletion led to four hemizygous genes in chromosome 7 in the Vd genome. D. Scatterplot between the number of SVs and ASE genes per Mb. E–F. Schematic representation of the gene structure for *PN003575* on two haplotypes in MF and the expression counts number for two alleles. The 303-bp deletion was identified by comparing the defined promoter regions between two haplotypes.

Through four assembled haplotypes from Vd and MF grapes, coupled with the PNT2T genome [50], we investigated gene loss and gain across distinct grapes. To reduce potential annotation errors, we employed the same gene annotation process as that used for the PNT2T genome. Based on the comparative genome analysis and genome-wide collinear blocks, we identified 27 122 genes acting as conserved genes in these grape haplotypes (Fig. 3b). We found that the gene number varied across different haplotypes, ranging from 2613 to 3430. At the same time, the divergence between two haplotypes from the MF genome was higher than the Vd genome, which was consistent with the higher variable gene number (Fig. S5). Furthermore, we used homology clustering to analyze gene families. We classified these gene families into core families, soft-core families, and specific families within the genome. Notably, 16 920 core gene families were identified, with variable soft-core gene families ranging from 304 to 564 (Fig. 3a). PNT2T exhibited the highest variability in gene families, potentially associated with the near-homozygous genetic background. We identified that the specific gene families across five haplotypes ranged from 76 to 122.

Previous studies revealed that SVs are related to the presence and absence of genes in the genome [51]. We focused on heterozygous haplotype-specific insertions or deletions in Vd and MF genomes, respectively. Notably, extensive SVs could impact the gene structure, rendering them hemizygous. For instance, an 82-kb region on chromosome 7 in the wild genome contained four hemizygous genes (Fig. 3c), this phenomenon also has been found in MF (Fig. S6). Approximately 10.4% genomic regions in cultivated grape genome were influenced by these heterozygous variations and resulted in 3468 (10.9%) hemizygous genes and a lower proportion of hemizygous genes detected in the wild grape genome (6.7% hemizygous genes) (Fig. S7).

SVs manifesting in the promoter region may influence the expression level, thereby contributing to phenotypes [52]. Transcriptome data from fruits were collected and used for identifying allele-specific expression (ASE) genes between two haplotypes in wild and cultivated grapes, respectively (Table S8). We conducted a correlation analysis between ASE and heterozygous SVs situated within the promoter region (2-kb) (Fig. 3d). The outcomes revealed notable positive correlations in both wild and cultivated grape genomes (*P-*value <7.7e-12). In addition, the cultivated grape genome exhibited more ASE genes compared to the wild grape. Specifically, our investigation identified two ASE genes in the cultivated grape genome: *PN003575* encoded a B-box domain protein and *PN020576* encoded an AUXIN RESPONSE FACTOR11 homologous protein (Fig. 3e, Fig. S8). Two distinct deletions (303 and 1034 bp) in their promoter regions might be associated with the different allele expression. The expression of the specific allele harboring a 303-bp deletion in the promoter region is markedly repressed (Fig. 3f).

**Table 1:** The genomic features of Vd and MF.

	Vd_H1	Vd_H2	MF_H1	MF_H2	PNT2T
Genome size (Mb)	518.48	513.30	489.74	486.96	494.87
GC content (%)	34.95	34.94	34.91	34.98	35.08
BUSCO (%)	98.4	98.0	98.4	98.4	98.5
Contig N50 (Mb)	26.50	26.13	24.09	24.06	26.90
Number of genes	31 711	31 368	33 351	33 153	37 534
Telomeres annotated	36/38	35/38	36/38	36/38	36/38
Centromeres annotated	19/19	19/19	19/19	19/19	19/19
The length of centromere (Mb)	23.22	27.39	18.89	28.49	27.26
TE length ratio in the genome (%)	49.56	49.15	48.16	47.71	48.73
The length of TE (Mb)	257.0	252.3	235.8	232.2	241.1

### R gene landscape in the grape genome

**Figure 4:**
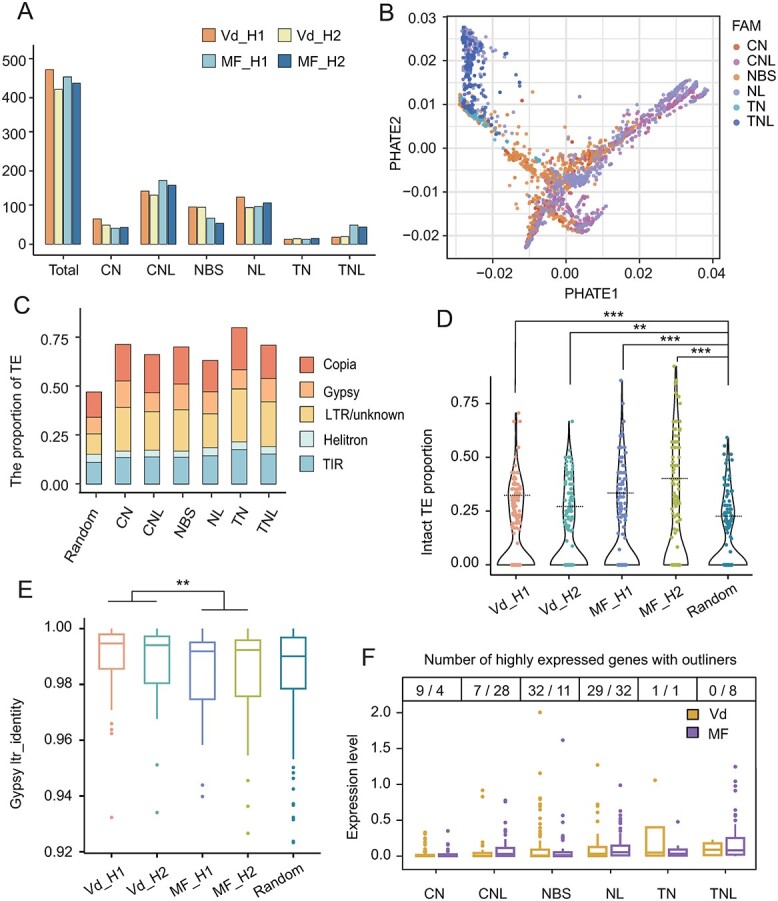
Distribution and pattern of R genes in wild and cultivated grapes. A. The number of six categories R gene on four newly assembled haplotypes. B. PCA analysis of six category R gene protein sequences. C. The proportion of TEs in genomic region (50-kb) nears R gene. The five classifications of TE were highlighted, and the 1% randomly selected genes were used as genome background level (random). D. The proportion of intact LTR-type TEs in genomic region (50-kb) nears R gene. E. The identify value of LTRs in the intact LTR/Gypsy. F. The number of highly expressed R genes in Vd and MF genomes with outliers.

Plant resistance (R) genes are integral components of disease-associated genes that exhibit considerable variation both within and between species [53]. The wild grape Vd demonstrated stronger resistance compared to the cultivated grape MF [48]. To assess the R genes in grape genomes, we identify genome-wide R genes across eight major categories (CN, CNL, NBS, NL, TN, TNL, RN, and RNL) (see Methods). These categories were established based on the single or combinations of protein domains. To validate the classification of R genes, we analyzed high-dimensional features derived from the protein sequence (Fig. 4b). Our analysis identified 424–478 R genes in four haplotypes from grape Vd and MF genomes (Fig. 4a, Table S9). Notably, the CNL, NBS, and NL gene categories constituted an average of 76.5% of R genes in these haplotypes, whereas RNL and RN categories were not included because they contain no more than three copies per haplotype. Although the total count of R genes in different haplotypes showed proximity, only 73% of homologous genes with collinear relationships were identified among the four haplotypes, indicating a considerable dynamic nature of R genes. This dynamism was evident not only between distinct genomes (e.g. MF and Vd), but also within different haplotypes of the same individual. We found that 84% of R genes in the Vd genome are biallelic, compared with 56% in the MF genome. Overall, our analysis sheds light on the dynamism of R genes within wild and cultivated grape genomes.

To gain insight into the landscape of R genes in grape genomes, we investigated the enrichment of TEs [54] within a ±50-kb region surrounding the R genes. We randomly selected genes to establish the genome background. Compared to the genome-wide background, we observed a significant enrichment of 74.2% of TEs near R genes across all six categories (*P* < 0.001) (Fig. 4c, Fig. S9, and Table S10). The type II transposons (LTR/Copia and LTR/Gypsy) play an important role, leading to an increase in the proportion of TEs.

Furthermore, we computed the proportion of intact long terminal repeat (LTR)-type elements surrounding R genes across four haplotypes. The results revealed a significantly higher proportion (*P* < 0.01) of intact TE enrichment compared to the genome-wide level (Fig. 4d). In addition, we calculated the identity of LTR in associated intact TEs. Higher identity values indicate more recent TE activity. Our findings demonstrated that the identity values of intact LTR/Gypsy and LTR/Copia around R genes exceeds the genome background level (Fig. 4e, Fig. S10). Interestingly, R genes in the wild Vd grape exhibited higher identity compared to the cultivated MF grape in both intact LTR/Gypsy and LTR/Copia. In addition, we integrated transcriptome data and performed expression analysis of the genome-wide R genes from four haplotypes (Fig. 4f). Most R genes exhibited low expression levels, and we defined genes with significantly higher expression as outliers. Compared to cultivated grapes, we found that NBS-type R genes in the wild Vd grape showed more outlier expression.

### Evolution of R gene clusters

**Figure 5:**
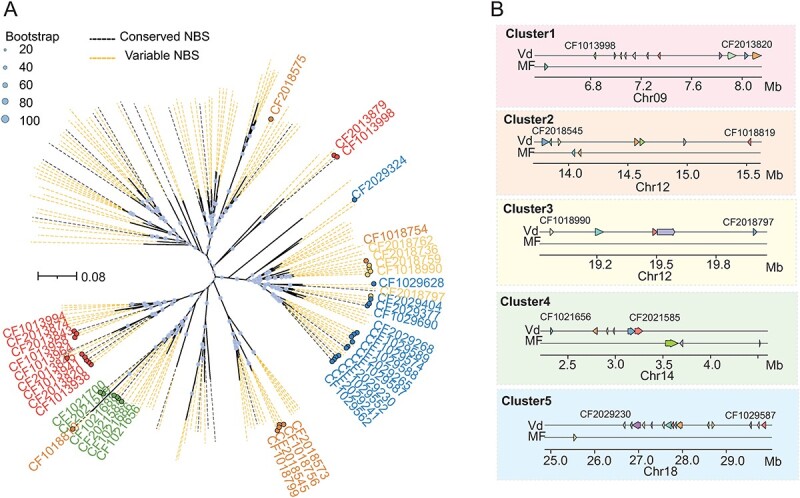
R gene cluster analysis of NBS genes. A. Phylogenetic tree of all NBS-type genes in the Vd genome. The variable NBS-type genes in four haplotypes were highlighted and NBS-type genes from five specific clusters were distinguished. B. Five specific NBS-type R gene clusters in the Vd genome and the syntenic regions in the MF genome.

To further investigate the genomic patterns of R genes and their duplication in grapes, we constructed phylogenetic trees based on the protein sequences of different type R genes (Fig. S11–S12). We observed a prominent clustering effect within the same chromosome (Table S11). We also identified 35 R gene clusters across four haplotypes, mainly in the CNL, NL, and NBS families, with NBS-type R genes showing the highest number of clusters (Fig. S13–S18). These results suggested that 78% of clusters were detected in both wild and cultivated grape genomes; however, within these clusters, only 45% showed homology among four haplotypes. In particular, the phylogeny analysis revealed that 85% of NBS-type R genes were variable, with at least one haplotype lacking the homologous gene (Fig. 5a).

Our findings suggest that genes from the same cluster were distributed in closer phylogenetic branches. To further elucidate the pattern of R gene clusters in the grape genome, we compared the distribution of R gene clusters between wild and cultivated grapes. We identified 59 and 48 clusters in the Vd and MF genomes, respectively. To compare the clusters from different haplotypes, we used the PNT2T genome as a reference and mapped these clusters. We found significant differences in the number of NBS-type R gene clusters between cultivated and wild grapes (Fig. S15). There were five clusters (Cluster 1–5) enriched with multiple NBS-type R genes in the wild Vd grape, whereas in the corresponding positions, without cluster in the cultivated grape genome (Fig. 5b). These clusters were distributed across chromosomes 9, 12, 14, and 18, with chromosome 12 containing two clusters, spanning 2.0 (13.5–15.5 Mb) and 1.2 Mb (18.9–20.1 Mb), respectively. Notably, Cluster 1 in the wild Vd grape had 11 NBS-type R genes, while there was only one gene in the cultivated MF grape at the corresponding location.

### Improving genetic mapping of R genes involved in white rot resistance

**Figure 6:**
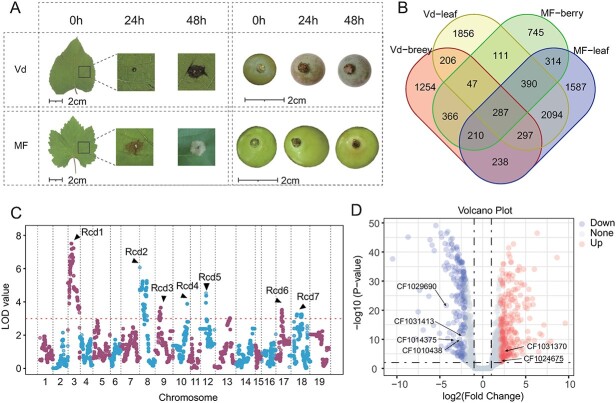
Genetic mapping of white rot resistance in the Vd genome. A. Schematic diagram of transcriptome period, organization, and phenotypes in the of *C. diplodie* inoculation experiment. B. Venn diagram of genes with elevated expression in different varieties and tissues after inoculation. C. QTL mapping for the Rcd profiles of F_1_ progeny derived from the genetic population. D. Volcano plot of the significant differential expression of *V. davidii* leaves after inoculation with *C. diplodie.*

To unravel the genetic architecture of R genes implicated in the immune response of grapes, we collected expression data from *Coniella diplodiella* (Speg.) Sacc. infection, a pathogen of grape white rot [48]. The expression dataset encompassed experiments conducted on leaves and fruits of both wild Vd and cultivated MF grapes, with and without inoculation of white rot disease. Time series samples were collected at 24 and 48 h postinfection. The phenotypic responses to white rot disease exhibited marked distinctions, with Vd demonstrating significantly higher resistance than MF (Fig. 6a). To assess the candidate differentially expressed genes (DEGs) to resistance, we aligned these expression data to the Vd reference genome and compared expression profiles between infected samples at 24 and 48 h and untreated samples (0 h). The resulting joint expression matrix was used for downstream analysis, revealing 2905, 5288, 2470, and 5417 genes with significantly changed expression in fruits and leaves of Vd and MF (Vd-berry, Vd-leaf, MF-berry, and MF-leaf) (logFC ≥2) (Fig. 6b). Notably, the number of differentially expressed genes with increased expression in leaves was approximately twice that in fruits. Among these, 837 genes exhibited significant expression difference in both fruits and leaves of Vd, while 1201 genes showed significant expression difference in both fruits and leaves of MF. We also identified 550 genes with specifically increased expression in the wild Vd grape leaves but not induced in the cultivar MF. Enrichment analysis of these genes in Gene Ontology (GO) functional terms revealed associations with biological processes (Fig. S19).

Furthermore, we collected previously sequenced data from the F_1_ progeny derived from Vd and MF and aligned these reads to the Vd genome to construct a high-density genetic linkage map. The integration of 3 years of phenotypic data enabled the mapping of quantitative trait loci (QTLs) associated with the resistance to *C. diplodie* (Rcd) (Fig. 6c). As expected, the published locus Rcd1 exhibited the highest LOD values and encompassed a notably significant genomic region. In addition to the four previously identified QTLs (Rcd1, Rcd2, Rcd5, and Rcd7) [48], our investigation unveiled three novel QTLs situated on chromosomes 9, 10, and 17 (Rcd3, Rcd4, and Rcd6). These loci provided a moderate explanation for the phenotype, with Phenotype Variance Explanation (PVE) ranging from 5% to 10%. The cumulative genomic length of these significant regions amounted to 58.3 Mb, constituting 11.3% of the entire genome. Within these regions, we identified 72 NBS genes, representing 40.7% of all NBS-type genes in wild grape Vd genome (Fig. S20). We investigated the QTL-associated NBS-type genes and analyzed their expression matrix, identifying six genes with significantly different expression levels (Fig. 6d, Fig. S21, and Table S14). Four NBS-type R genes exhibited significant downregulation after inoculation, while two genes demonstrated significant upregulation. Among them, *CF1069290* showed the most pronounced downregulation fold change. Our results provide potential evidence for a narrowed set of candidate R genes involved in the response to white rot tolerance within the wild grape genome.

## Discussion

The grape genome has undergone significant improvement, achieving gapless assembly spanning from T2T in the PNT2T version [50], marking a major advance in grape breeding. The availability of high-quality genome assemblies is instrumental in expanding the genetic diversity of grape breeding programs. By incorporating assemblies from diverse grapevine cultivars and wild relatives, breeders can access a wider pool of genetic variation. One of the most promising applications of wild grape genome assemblies lies in the identification of disease-resistant loci. The utilization of wild germplasm resources is conducive to improving the effectiveness of grape breeding. Researchers are paying great attention to the genetic information of wild grapes. Noé Cochetel et al. used Illumina HiSeq 4000 sequencing technology to assemble the genomes of 9 American wild grape species, including *Vitis acerifolia*, *Vitis aestivalis*, and *Vitis arizonica*, but did not achieve T2T gap-free assembly quality [28]. The assembly of four Chinese wild grape genomes has been completed; however, genotyping was not fully achieved, and gaps were present in the genomes of *V. amurensis* [29], *V. adenoclada* [30], and *V. heyneana* [31], except the genome of *V. retordii* [16]. *Vitis davidii* is a typical representative of disease-resistant wild grape resources in southeastern China, characterized by excellent disease resistance [55]. Therefore, the assembly of wild grape *V. davidii* will be beneficial for exploring the genetic basis of resistance and further guiding grape breeding.

Our assembly of the wild grape *V. davidii* provides insights into the landscape and genomic patterns of resistance loci in the grape genome. R genes play a crucial role in the immune defense responses of plants by directly recognizing pathogen effectors [56]. The contraction and expansion of R genes are influenced, in part, by selection pressures to adapt to complex environments [57,58]. Through comparisons of multiple haplotypes from wild and cultivated grape genomes, we found a similar number of R genes. Interestingly, our examination showed that 60% of R genes in the grape genome exhibited variability across grape genomes, even when comparing paired haplotypes within a single sample. The dynamic nature of R genes contributes to the gene pool essential for constructing intricate resistance networks during plant evolution [59]. In our exploration, we traced the enrichment of TEs in regions proximal to R genes.

SVs have the potential to drive phenotypic evolution, with one possible mechanism acting as regulators in promoters, influencing gene expression [60]. Previously, Kim et al. [61] and Maestri et al. [25] revealed the evolution of plant disease-resistance genes in pepper plants and their expansion was associated with retroduplication. The association between variable genes in the grape genome and distinct SVs in different species or haplotypes also highlights the impact of SVs on gene copy variation. SVs may insert into the R gene structure, thereby disrupting the R gene. Large SVs covering the entire structure of the R gene have been reported in crop genomes [62–64]. Gene duplication leads to the production of redundant copies of the R gene in the grape genomes [63]. The duplicated R genes can be retained or lost during evolution, thus facilitating rapid evolution against strains with pathogenic factors. Similarly, research by Guan et al. [65] suggested that genomic SVs drive functional discoveries in peach genomes. Moreover, the role of LTR-type TEs, as indicated by Rispe et al. [66], significantly impacts R genes evolution. This finding aligns with the study conducted by Lu et al. [67], which investigated the evolution of wheat sharp eyespot *Rhizoctonia cerealis*.

The arrangement of R genes primarily in clusters, exhibiting exceptionally high protein homology within each cluster, marks expansion events in gene family evolution [68]. This clustering suggests potential collaborative interactions among the gene products of paired and larger clusters during immune activation. These five specific NBS-type R gene clusters were enriched in the genome of Vd, which might be related to synergistic modulation of immune responses. It is common for different genes within these clusters to undergo subfunctionalization in response to specific pathogenic factors or even lose function over time.

The phase-resolved genomics has opened new possibilities for identifying founder haplotypes and tracing them in breeding populations, as demonstrated in the study by Minamikawa et al. [69]. These studies collectively demonstrate the pivotal role of the T2T reference genome in advancing molecular breeding applications, gene function discovery, and understanding genome evolution, thus contributing to the improvement of crop breeding and agriculture. After replacing the reference genome with the newly generated assemblies of *V. davidii*, we performed genetic mapping using previously published populations [48] to investigate novel disease-resistant loci. Our investigation newly identifies three QTLs situated on chromosomes 9, 10, and 17 (Rcd3, Rcd4, and Rcd6). Several similar genomics-driven projects have been established. For example, the FruitBreedomics project has highlighted the importance of bridging the gap between molecular genetics research and its application in breeding, thereby increasing breeding efficiency in fruit tree crops [70]. Going forward, combining these new QTLs from *V. davidii* with molecular marker-assisted breeding may significantly accelerate grape resistance breeding.

## Materials and methods

### Plant materials and genome sequencing

A wild grape *V. davidii* Föex (0940) and a cultivated grape *V. vinifera* L. Manicure Finger, materials conserved at the National Repository for Grapevine and Peach (Zhengzhou, China). Fresh leaves served as sequencing materials. We collected these leaves and subsequently frozen in liquid nitrogen. These leaves are then ground in a mortar at a low temperature for genomic DNA extraction. We extracted genomic DNA based on the improved CTAB method. Two genomic sequencing methods, HiFi and Hi-C sequencing, were employed in this study. For the Hi-C libraries, we constructed by digestion with the four-cutter restriction enzyme MboI. The prepared libraries were sequenced on the Illumina HiSeq X Ten platform (San Diego, CA, USA) using 150-bp paired mode. For HiFi sequencing, HiFi reads were sequenced on the PacBio Sequel II platform using the circular consensus sequencing (CCS) mode. The raw sequencing data was preprocessed using SMRTlink software, which included removing adapters and correcting subreads to generate high-quality HiFi sequences for further analysis.

The PNT2T genome [50] as a reference, which was sequenced using PacBio HiFi and fully annotated. Additionally, collected RNA-seq data from NCBI for 0, 24, and 48 h after inoculation with live white rot bacteria on leaves [48] and berries (SRR shown in Table S12).

### Genome assembly

We combined HiFi and Hi-C data to assemble the genomes of two grape accessions using the Hifiasm [71] program with default parameters. For each accession, we obtain two separated contig-level haplotype assemblies. The heterozygosity was investigated using a k-mer-based approach integrated in GenomeScope [72] v2.0. To improve the assembly, the Hi-C data were utilized to anchor and remove certain short contigs (<100 kb). The program RagTag [73] was employed to determine the approximate order of contigs on chromosomes, with PNT2T serving as the reference genome. The Hi-C reads were further used to anchor all contigs using Juicer [74] v1.5, followed by a 3D-DNA [75] scaffolding pipeline. Subsequently, we manually adjusted these genome assemblies using Juicebox [76,77] before rerunning 3D-DNA to obtain the genome at the chromosome level. The completeness of four haplotypes was evaluated using BUSCOs [78] with the embryophyta_odb10 database, while the genome continuity was assessed by calculating the length of contig N50.

### Identification of telomeres and centromeres

We used the telomere pipeline developed by TIDK [79] v.0.2.0 to identify the repeated unit of telomeres. As a result, we identify telomeric sequences characterized by the unit 5’-CCCATTT and 3′-TTTAGGG. The telomere peak line was visualized using R scripts based on rapid statistics of telomeres. Tandem repeat annotation was performed using TRF [80] v4.09, and the results were merged using the TRF2GFF script. Data statistics was completed by extracting information using the awk command in the Linux system and visualizing the results in Integrative Genomics Viewer [81] (IGV, v2.12). The TRF program was used to scan tandem repeats in the genome. According to *V. vinifera* centromeric repeat sequences, we confirmed a 107-nucleotide monomer (AGTACCGAAAAAGGGTCGAATCAGTGTGAGTACCGAAAAATGGTAGAATCCGGGCGAGTACCGGGAAAAGGTAGAATCCGTGCGAGTATCGAAAAACTGTCCGGGCG) in the centromere region of each chromosome of two assemblies.

### Genome annotation

The gene structure was annotated based on evidence from expression data and homologous protein. These expression data were aligned to the softmasked assembly using Hisat2 [82] v2.10.2 and assembled into transcripts with StringTie [83] v1.3.0. To identify genes, transcripts were initially searched using UniProt [84] as supporting evidence. For genome annotation, we primarily utilized the multiple programs including MAKER [85], SNAP [86], and PASA [87]. We constructed a preliminary gene model, followed by further searching with AUGUSTUS [88] v3.4.0. Genes located in duplicated regions, with CDS regions shorter than 90 or lacking evidence, were filtered out. Additionally, we checked missing genes and refined gene models based on alternative splicing analysis. Consequently, we validated these gene structures using the Hidden Markov Models based on the Pfam [89] database and filtered the low-confidence gene structure.

To identify genomic TEs, we annotated repeated sequences by the Extensive *de novo* TE Annotator [90] (EDTA v2.1.0) and categorized into four major clades including LTR, terminal inverted repeat (TIR), long interspersed nuclear element (LINE) repeats, and Helitron-like DNA transposons. For the downstream annotation, we masked repeat element sequences using the program RepeatMasker [91] v4.1.2.

### Resistance gene identification and validation

We detected genome-wide R genes based on the domain and motif structures using four haplotypes from two grape accessions. The RGAugury [92] v.2.2 pipeline and NLRtracker [93] were used to identify the putative nucleotide-binding site (NB-ARC) domain-encoding genes into eight subgroups: TN (Toll/interleukin-1 receptor (TIR) and NB-ARC), CN (coiled-coil (CC) and NB-ARC), NL (NB-ARC and leucine rich repeat (LRR)), CNL (CC, NB-ARC, and LRR), NB (NB-ARC), TNL (TIR, NB-ARC, and LRR), RN (RPW8 and NB-ARC), and RNL (RPW8, NB-ARC, and LRR) (Table S13). To verify the trustworthiness of R gene, we generated features of R protein sequences using the program iFeatureOmega [94]. To the best of our knowledge, this program expands its functionality by integrating 15 feature analysis algorithms. Collected high-dimension features from composition of three collections: k-spaced amino acid pairs (CKSAAP), Dipeptide composition (DPC), and Conjoint k-spaced triad (KSCTriad). The high-dimension matrix was introduced to the program PHATE [95] for dimension reduction and used for validating of R genes.

We analyzed TE enrichment surrounding each R gene and classified the TEs according to their respective families. Search intervals were defined as 50 kb upstream of the gene start site and 50 kb downstream of the gene end site. Overlapping TE structures within these intervals were included in the enrichment analysis. To provide a genomic comparison, we randomly selected an equivalent number of non-R genes and analyzed their corresponding intervals of identical length, repeating this process 100 times. To evaluate the robustness of our findings, we employed a jackknife subsampling test (α = 0.05) and performed a Student’s *t*-test for statistical significance, which yielded the final *P*-value.

### Analysis of genomic collinearity

We used Minimap2 [96] v2.24 to align the haplotype sequences to the reference genome, and the resulting alignment BAM file was indexed and sorted using SAMtools [97] v1.11. Then, we deployed the Synteny and Rearrangement Identifier [98] (SyRI) to identify collinear orthologs and genomic large rearrangements between the two haplotypes of the Vd and MF genome. For visual interpretation of the data, the Plotsr [99] tool was used. Additionally, MUMmer [100] v4.0 was used for an in-depth comparison of the Vd genome with the PNT2T reference genome. We aligned genome sequences using nucmer (nucmer --mum) and filtered the results (delta-filter -i 95 -l 10 000). Subsequently, we visualized the comparison results in dot plots. These readable comparison data were extracted using the ‘show-coords -T -q -H’.

### The analysis of SVs and hemizygous genes

Using SVIM-asm [101] v1.0.3 to detect chromosomal SVs in Vd and MF, with PNT2T as the reference genome. First, the four haplotypes were compared to PNT2T using minimap2 to obtain bam files; second, SVs were detected using SVIM-asm. For each identified SV, we viewed it in IGV to confirm its authenticity. Survivors [102] v1.0.7 was used to integrate the SVs on the four haplotypes, obtaining the SV datasets of Vd and MF relative to the PNT2T reference genome. Only SVs supported by multiple genomes were included as potential SVs here. We combined the SV map with gene annotation to identify hemizygous genes in the Vd and MF genomes. As a result, we filtered heterozygous SVs and identified hemizygous genes according to whether there is overlap (>10%) between SV and gene coding regions.

### Phylogenetic tree and gene clusters

The phylogenetic tree was constructed using two methods. First, using MEGA [103] software (bootstrap = 1000) to construct a phylogenetic tree for all NBS sequences. The second phylogenetic tree was drawn in Python using the ETE Toolkit [104] for the NBS cluster sequences. Downloaded a gene family with an hmm structure from the Pfam database and used hmmserach software to predict conserved domains. A cluster was defined as a 1.0-Mb genomic region containing at least three homologous proteins.

### Inoculation treatment

We perform inoculation treatment at the National Repository for Grapevine in Zhengzhou, China, using *V. davidii* (0940) and Manicure Finger grape varieties. We punctured fruits pedicels from both grape varieties with a sterile needle. Fungal mycelium was manually exposed to the wound mediated by a potato dextrose agar (PGA) block. The treated fruits were incubated at 28°C. Samples were collected at 24 and 48 h postinoculation, and the uninfected fruits serving as the negative control. We also conduct three biological replicates, and all samples were utilized for subsequent expression analysis. The RNA-seq data of leaves inoculation treatment was collected from BioSample accession number PRJNA938012.

### Analysis of RNA-seq datasets

We filtered and trimmed the raw RNA-seq data using the FastQC [105] program. These filtered RNA-seq reads were aligned to the reference genome using the hisat2 program. Using the featureCounts [106] -p —countReadPairs function, the sam files of all periods were counted according to the gene ID order in the gtf file, and the results were converted to Transcripts per million (TPM) for data normalization. We estimated the allelic expression of genes by counting the reads at the heterozygous SNPs. For a gene, the allelic imbalance level was calculated as the mean of the read count. We profiled significant ASE using ‘Fold Change’ ≥2 between alleles and FDR-corrected *P*-values <0.05 calculated based on the EdgeR [107] package in *R*.

### Genetic map construction and QTL mapping

The Vd genome was indexed using the BWA [108] tool and the population-level whole-genome resequencing data was aligned to the Vd genome. The BWA-generated BAM files were examined using GATK [109] markdropped function, with the ‘remove_dropped’ option set to ‘true’. To enhance precision, the BAM files were sorted by coordinates using the ‘sort’ command and then merged the sorted BAM files to extract SNPs for each sample using the GTX [110] tool. The final SNP dataset was generated using the ‘GTX -joint’ mode. Quality control was conducted using the PLINK [111] tool with the ‘-geno 0.2 —maf 0.05’ parameters.

We filtered the mismatch variants and collected available marker datasets for the fine mapping in the F_1_ population. To increase the accuracy of the association, we selected markers that covered all progeny. These markers showing significant separation distortion (*P* < 0.001) were also filtered. The high-quality genetic markers were assigned to linkage groups integrating chromosome information. Each linkage group was organized using LepMap3 [112] software. We calculated the genetic distance following the Kosambi function.

R/qtl2 [113] was used for QTL analysis. We choose the method of Haley–Knott regression to detect potential QTLs. To make the results more reliable, we also performed a thousand permutations to determine the statistics. We identify a threshold value (LOD = 3.0) to be considered significant. PVE was estimated based on candidate loci, and confidence intervals were defined using 95% Bayesian credible intervals.

## Supplementary Material

Web_Material_uhae306

## Data Availability

All the raw sequencing data generated for this project have been deposited in the National Genomics Data Center (NGDC) Genome Sequence Archive (GSA) (https://ngdc.cncb.ac.cn/gsa/) with the number of CRA017609. The assembly and annotation as well as the sequences of centromeres and heterozygous regions have been deposited in Zenodo (https://zenodo.org/records/12671741).
