# Supplementary material for: Phased T2T genome assemblies facilitate the mining of disease-resistance genes in Vitis davidii
Source: Hortic Res. 2024 Nov 6;12(2):uhae306. doi: 10.1093/hr/uhae306 (PMC11817892; doi:10.1093/hr/uhae306)
Supplement: Web_Material_uhae306 [file web_material_uhae306.zip › Supplement_Figure.pdf]

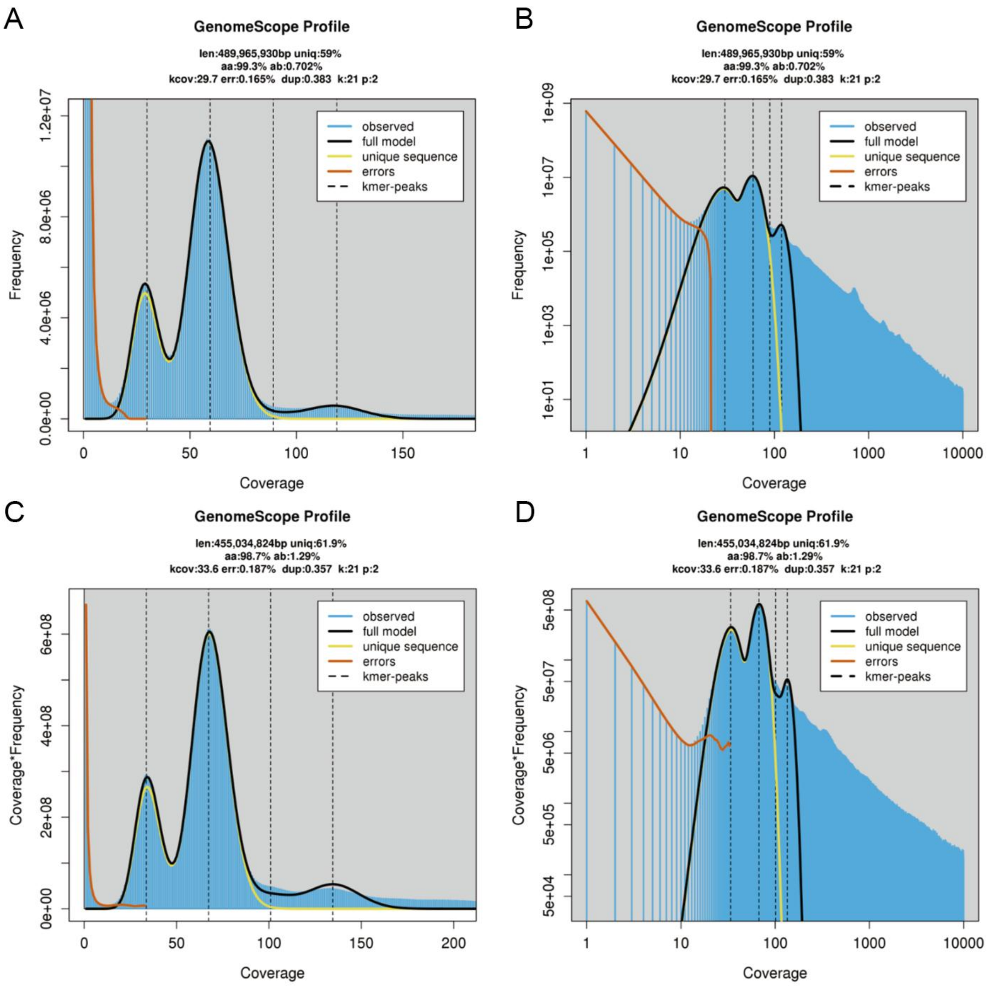

Figure S1. The genome size and heterozygosity of grape Vd (A B) and MF (C D) estimated by K-mers method.

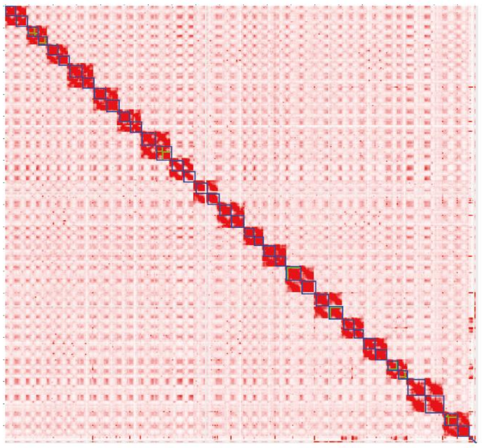

Figure S2. Hi–C interactions of chromosomes based on MF assembly data. The blue square represents pseudo-chromosomes/chromosomes, the green square represents different contigs. From top to bottom are chromosomes 1 to 19 respectively, and every two blue squares are the same chromosome of Haplotype 1 (upper left) and Haplotype 2 (lower right), respectively.

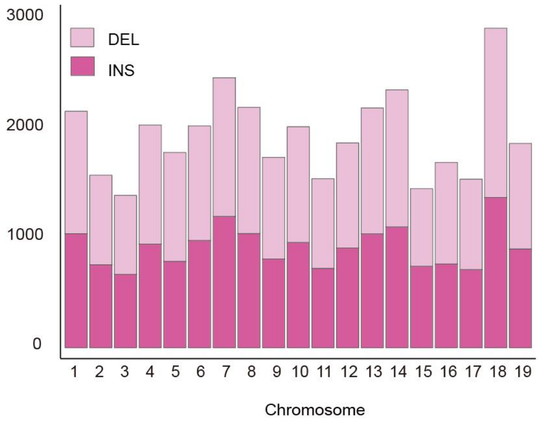

Figure S3. Genome-wide SV map between PNT2T and four haplotypes of Vd and MF.

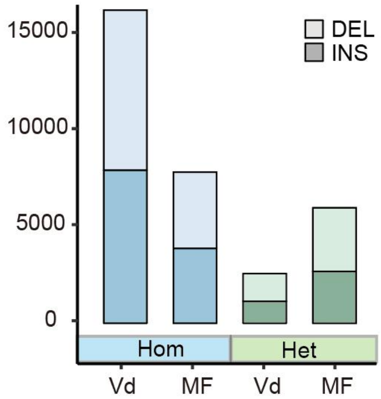

Figure S4. The number of homogeneous (Hom) and heterogeneous (Het) structural variations of DEL and INS between the two haplotypes of the Vd and MF genomes.

A

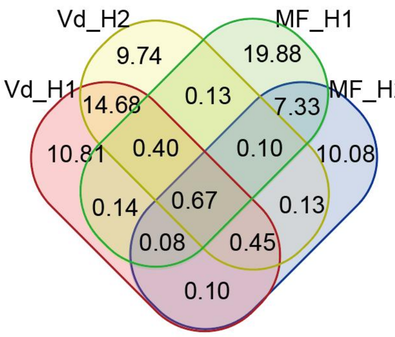

B

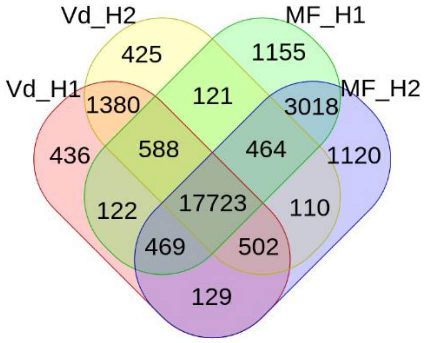

Figure S5. A. Length of characteristic gene segments between the four haplotypes of the Vd and MF genomes using PNT2T as the reference genome. B. The number of variable genes between the four haplotypes of the Vd and MF genomes using PNT2T as the reference genome.

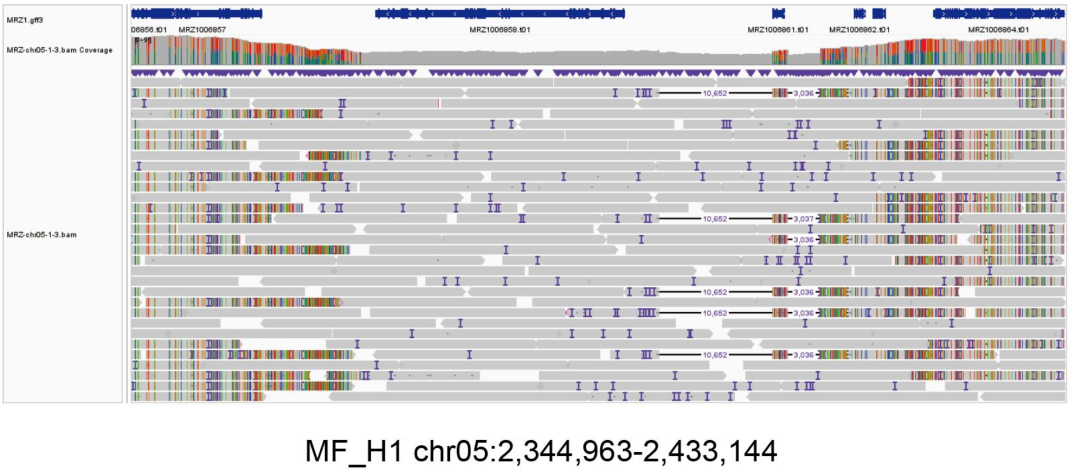

Figure S6. A heterozygous deletion led to a hemizygous gene in chromosome 5 in the MF genome.

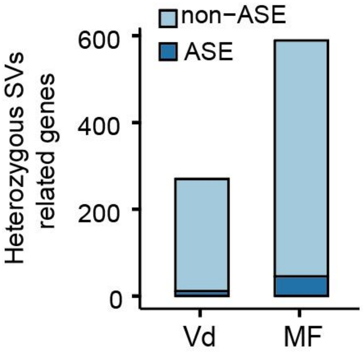

Figure S7. Number of ASE genes associated with heterozygous SVs between the two haplotypes of the Vd and MF genomes.

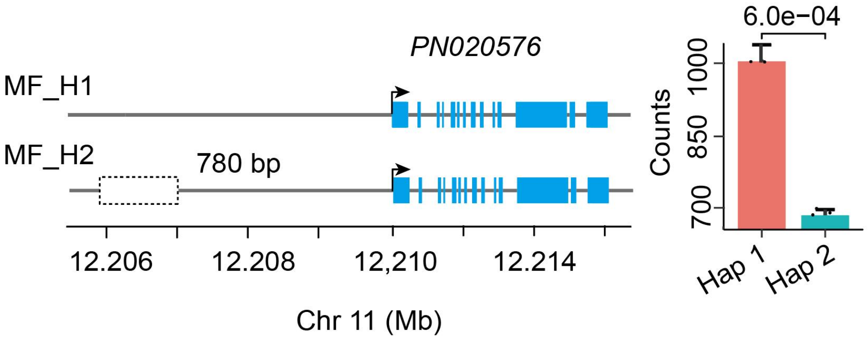

Figure S8. Schematic representation of the gene structure for *PN020576* on two Haps in MF and the expression counts number for two alleles.

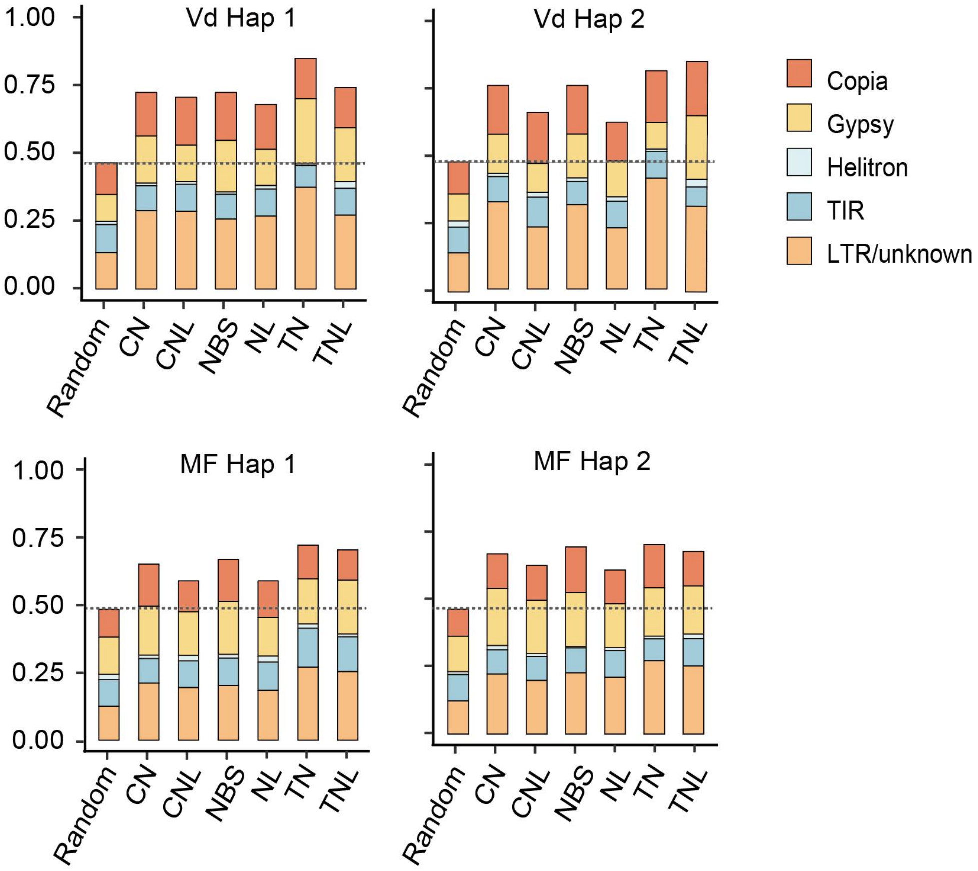

Figure S9. The different proportions of TE in six classes of R genes on the four haplotypes of the Vd and MF genomes. In the figure, Random means that 1000 genes were randomly selected from all genes.

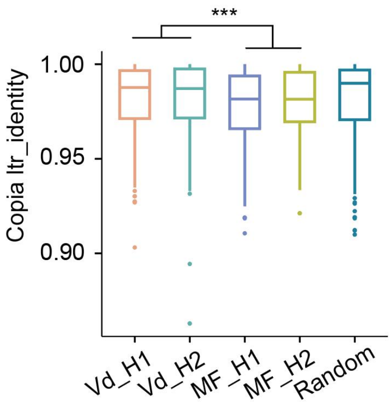

Figure S10. The identify value of long terminal repeats in the intact LTR/Copia.

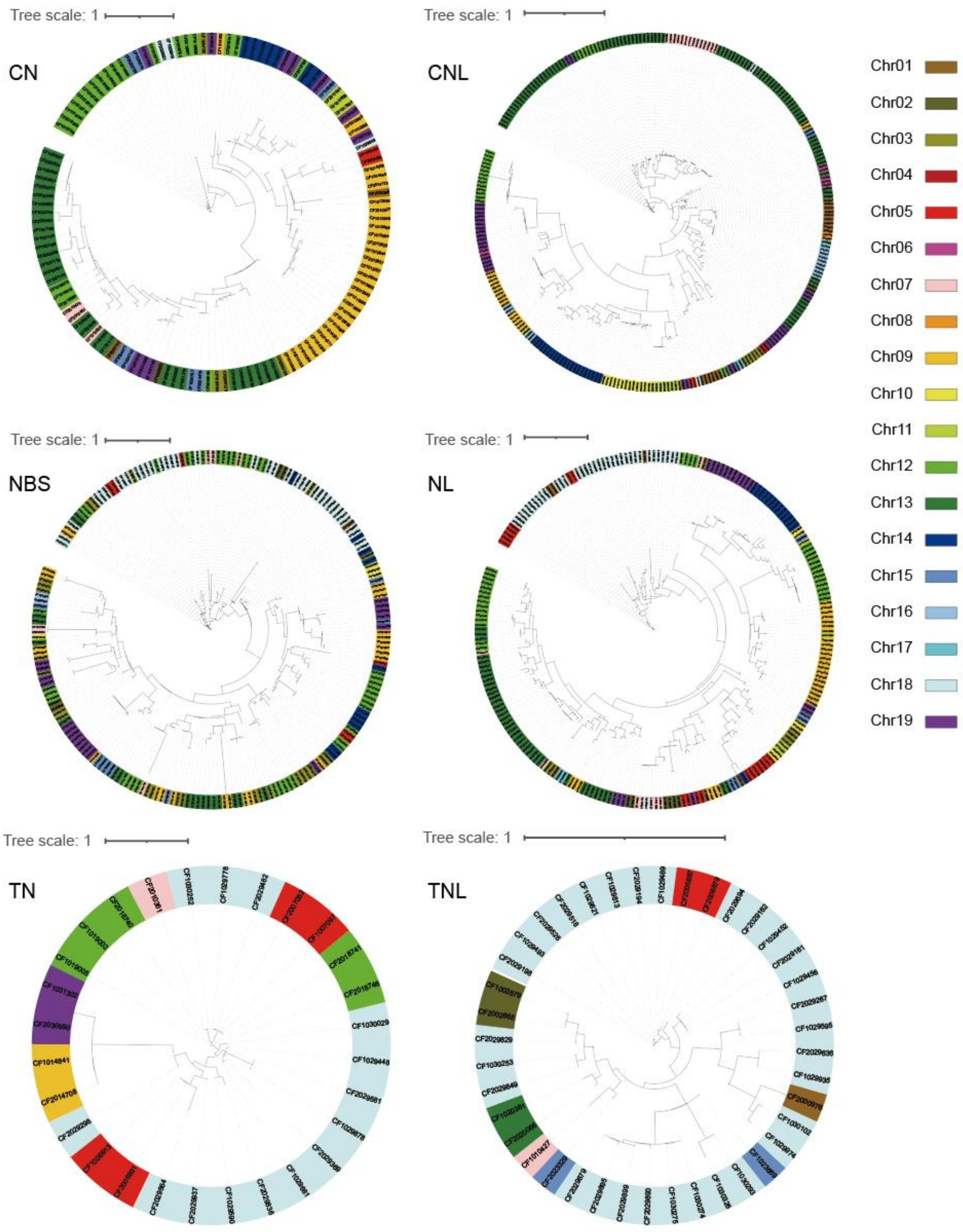

Figure S11. Evolutionary trees of the six classes of Vd's R genes and the color of the chromosome in which they are located are marked on the outer circle.

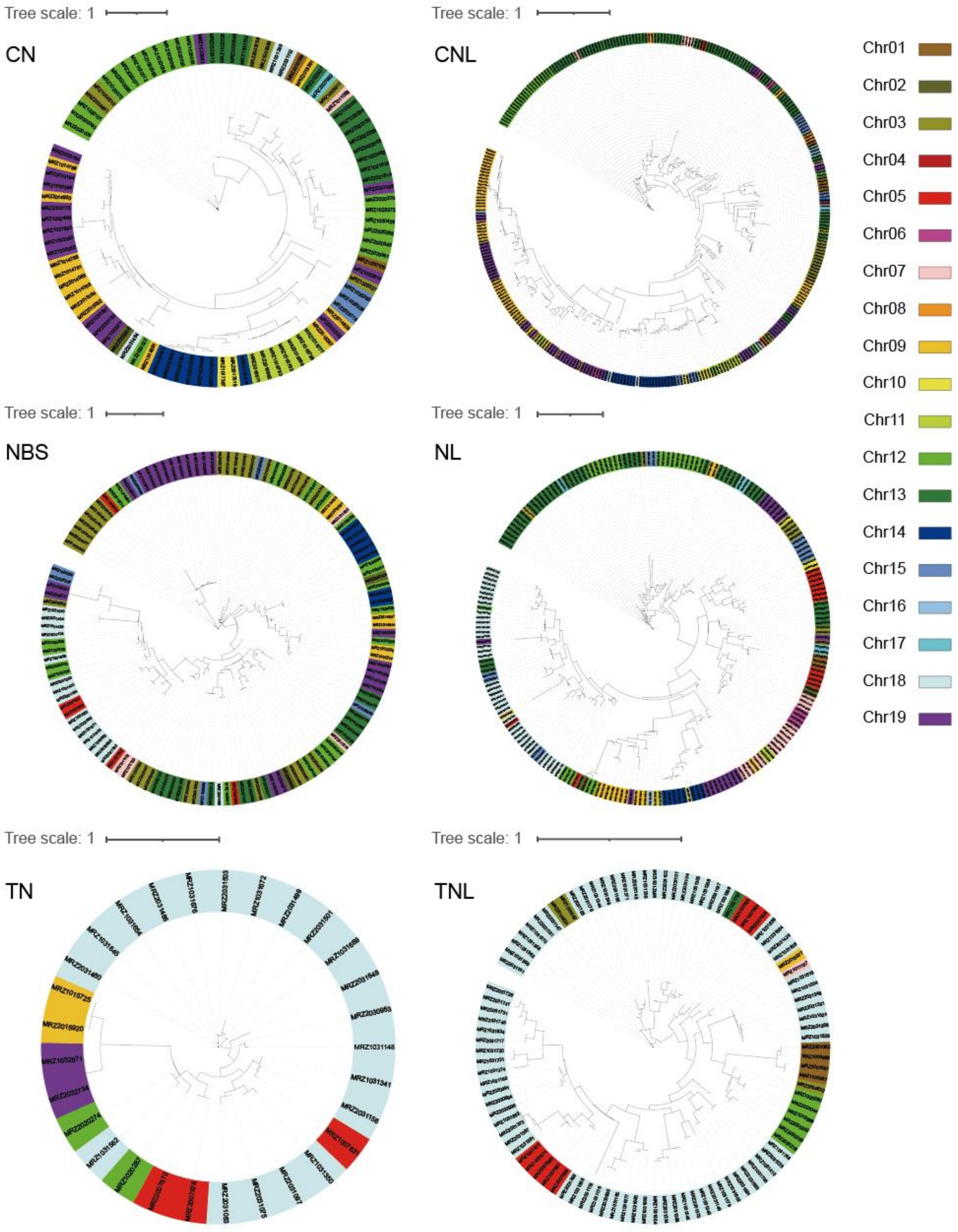

Figure S12 Evolutionary trees of the six classes of MF's R genes and the color of the chromosome in which they are located are marked on the outer circle.

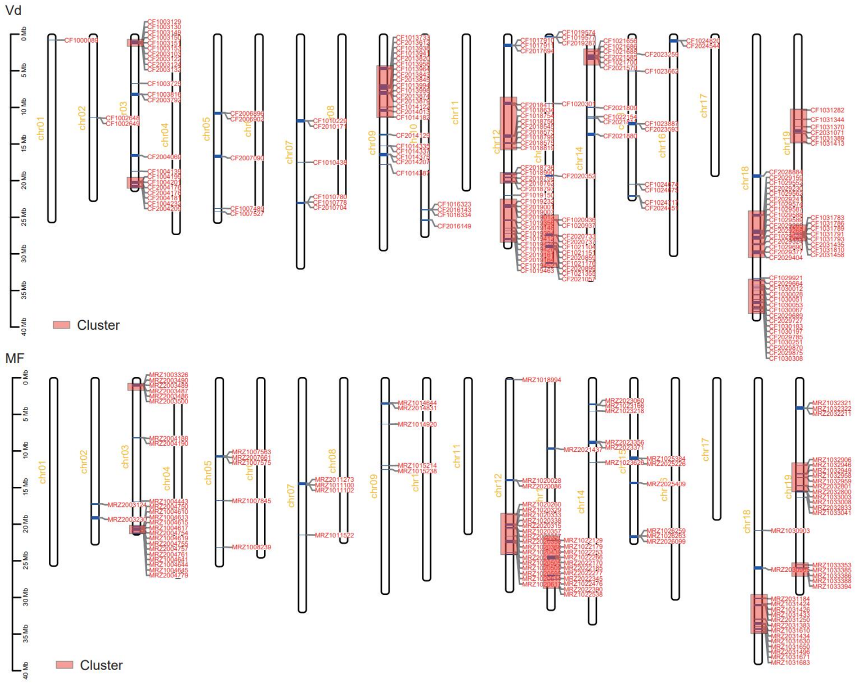

Figure S13. Location of Vd's and MF's CN-type R genes on chromosomes.

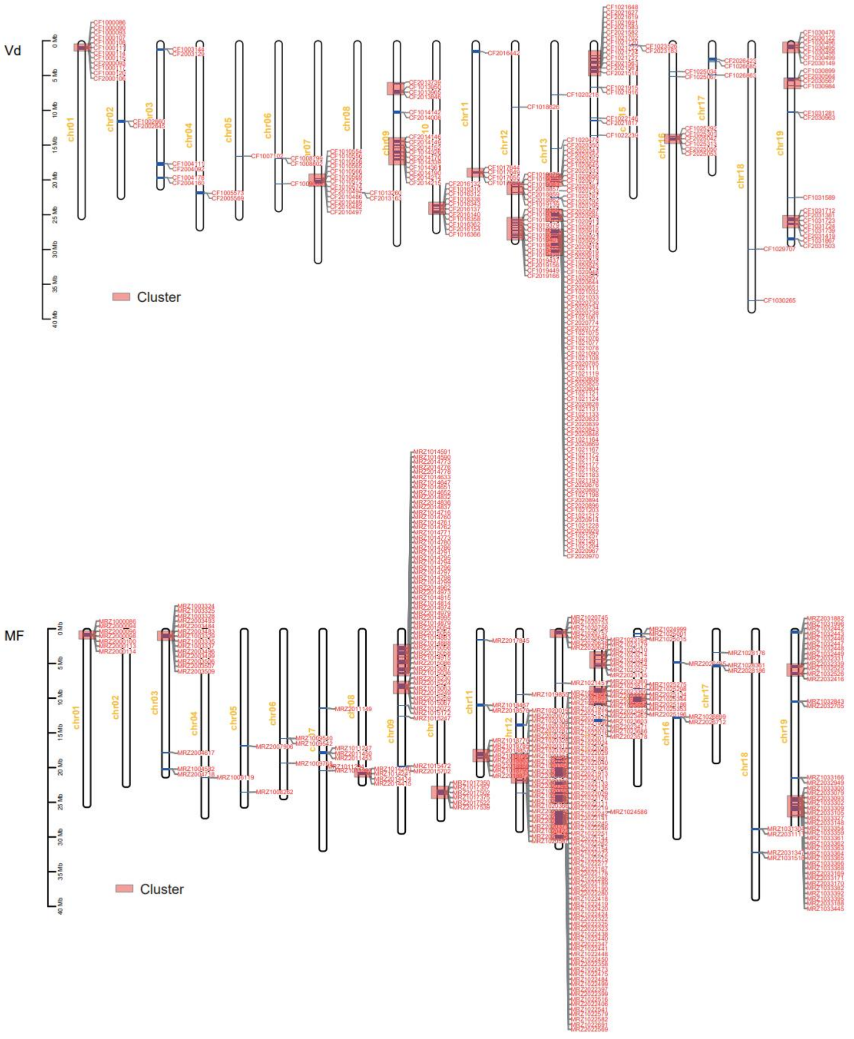

Figure S14. Location of Vd's and MF's CNL-type R genes on chromosomes.

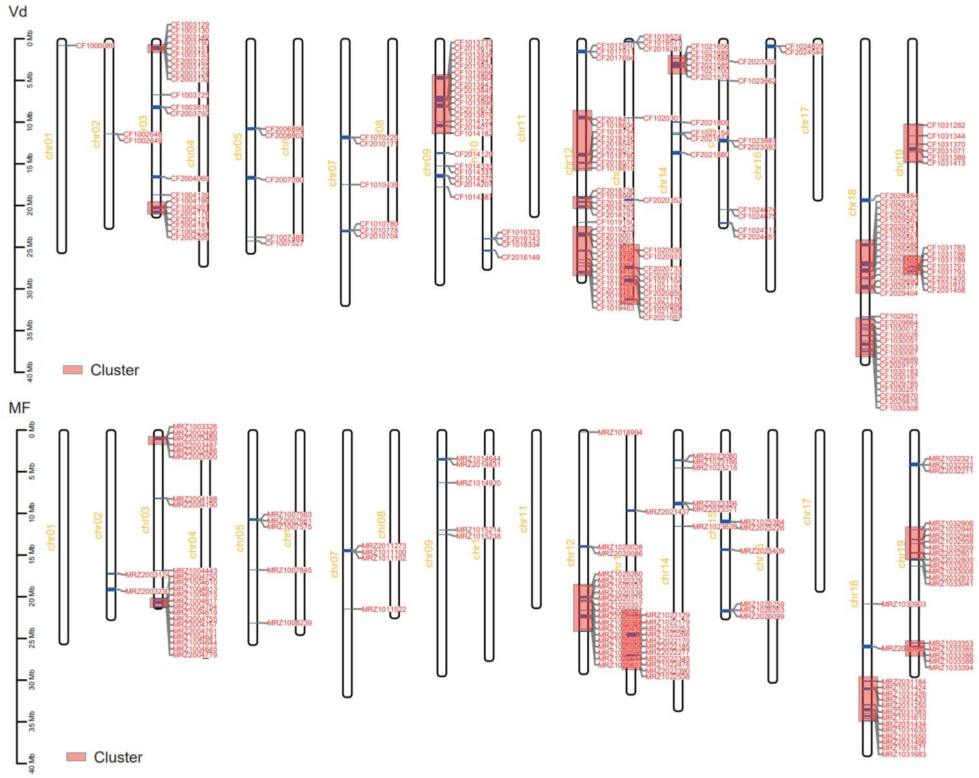

Figure S15. Location of Vd's and MF's NBS-type R genes on chromosomes.

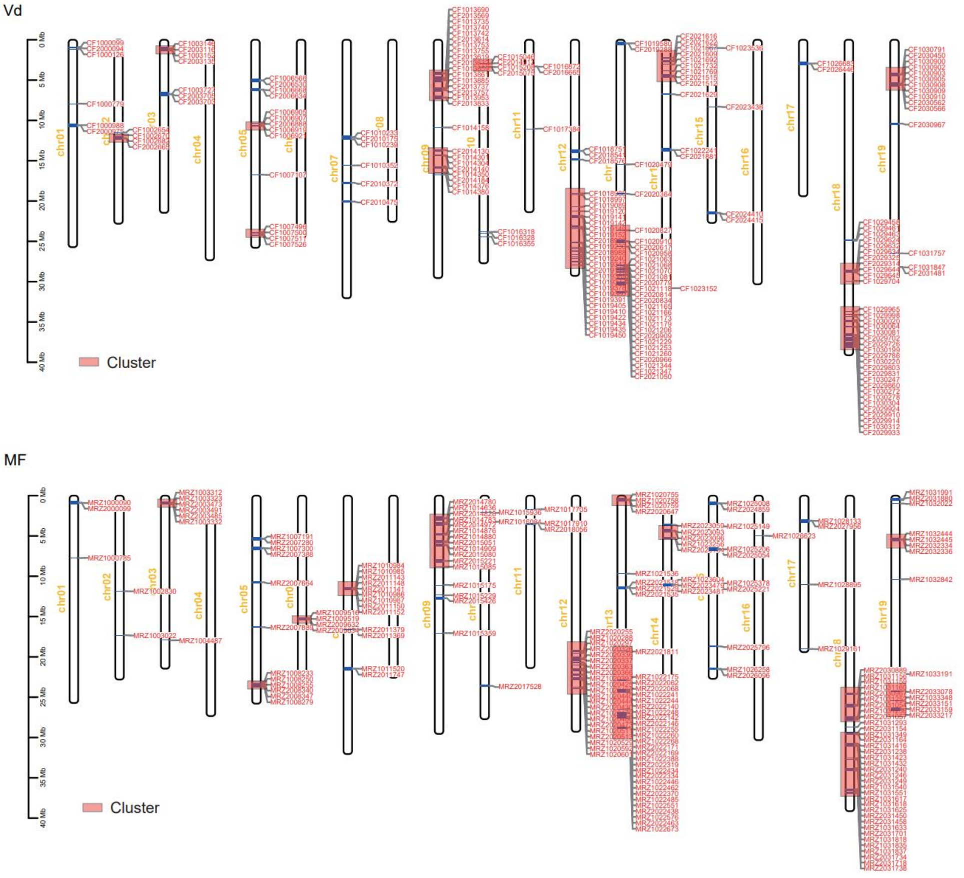

Figure S16. Location of Vd's and MF's NL-type R genes on chromosomes.

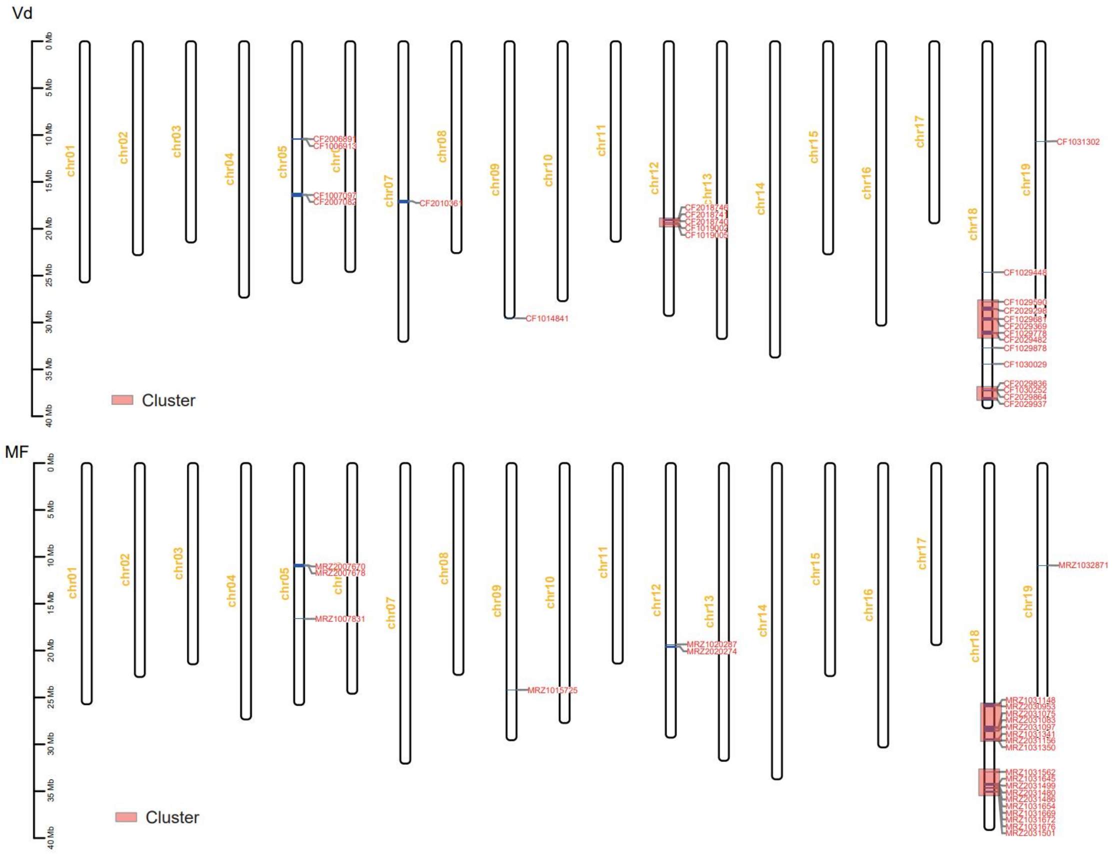

Figure S17. Location of Vd's and MF's TN-type R genes on chromosomes.

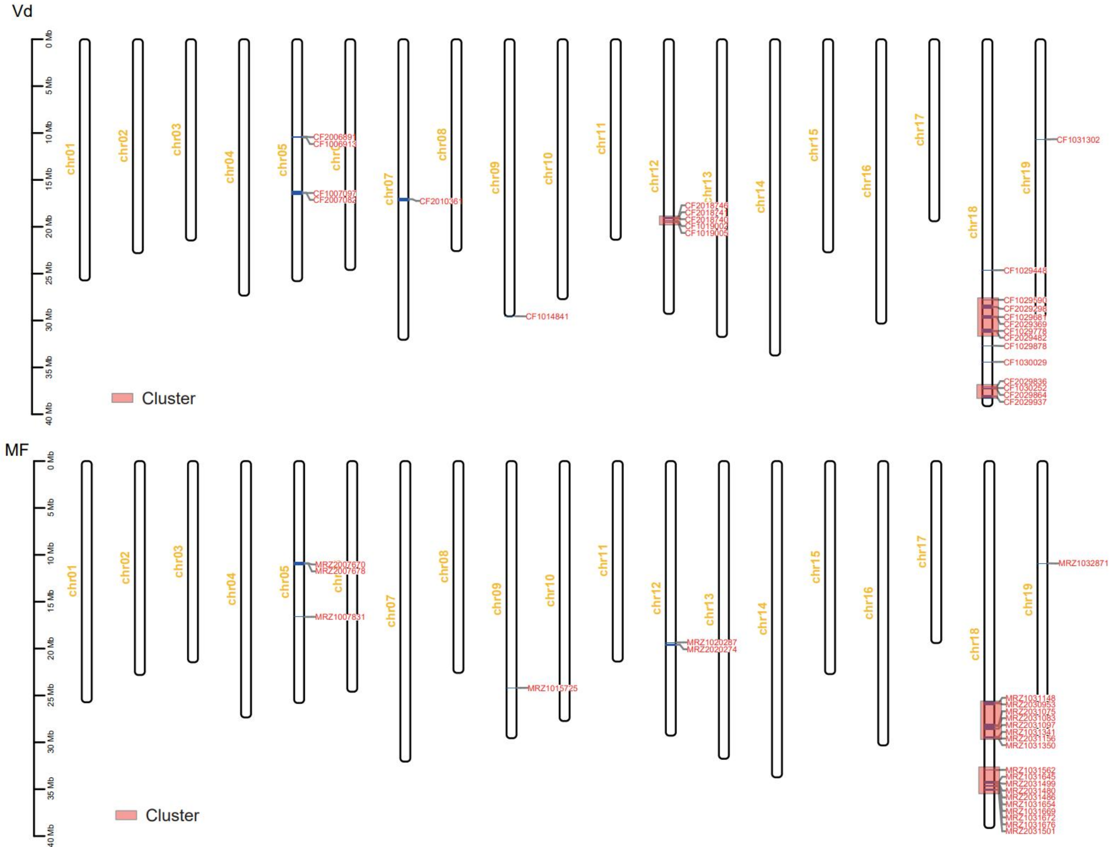

Figure S18. Location of Vd's and MF's TNL-type R genes on chromosomes.

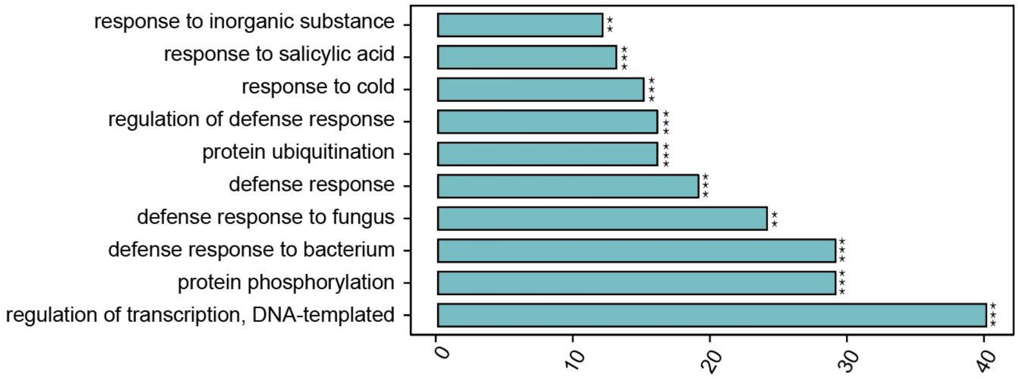

Figure S19. Enrichment analysis of 550 genes which were specifically increased expression in the wild Vd grape genome but not induced in the cultivated MF genome in Gene Ontology (GO) biological processes.

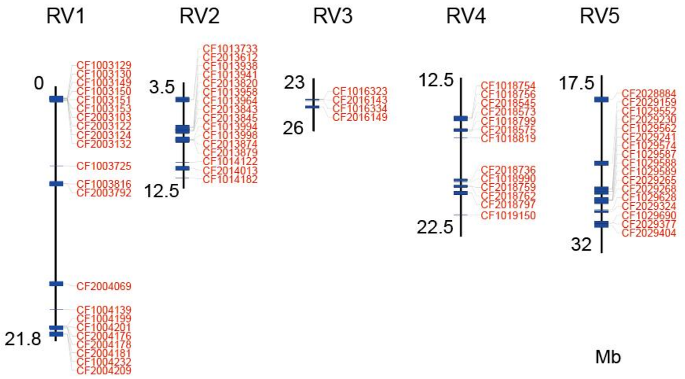

Figure S20. Regions containing NBS genes in the white rot disease's QTL.

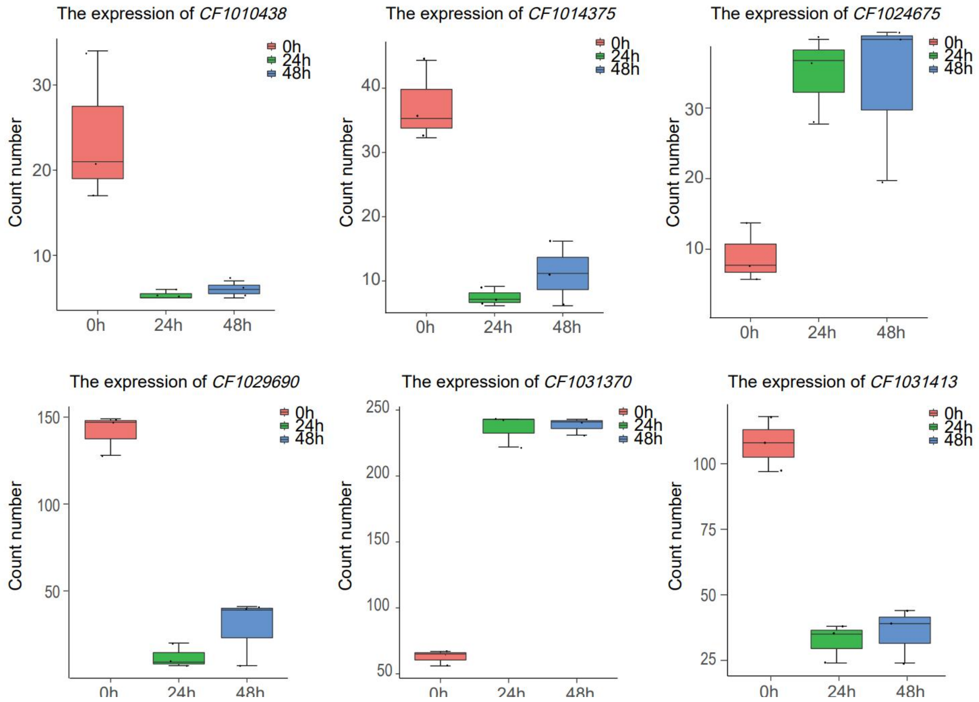

Figure S21. The expression of the six NBS-type genes which showed significant different expression after infection with *Coniella diplodiella* (Speg.) *Sacc.* at 0h, 24h, and 48h
